# Fifth Anniversary Meeting of the Provincial Medical and Surgical Association

**Published:** 1837-10

**Authors:** 


### FIFTH ANNIVERSARY MEETING OF THE PROVINCIAL MEDICAL AND SURGICAL ASSOCIATION,

*Held at Cheltenham, on Wednesday, the* 19*th, and Thursday, the* 20*th, days of July*, 1837.

BY AN ORIGINAL MEMBER OF THE ASSOCIATION.

There are many very respectable members of the profession who regard these anniversaries with indifference, as there are also many men who, whilst their love of science is unquestionable, regard the annual meetings of the British Association with a similar feeling. We are not of the number. Even if we were to grant, which we can by no means do, that the cause of science is very little advanced by these periodical meetings, we should contemplate them with pleasure, as affording opportunities for the cultivation of those liberal feelings, in the absence of which science itself loses half of its attractions. In whatever towns the British Association has held its meetings, science has experienced a local and salutary impulse; and, wherever the Medical and Surgical Association has assembled, it has probably done something towards harmonizing the resident members of the profession. The rivalry of those who advocate different views is by nothing more mitigated than by a personal introduction of the rivals. The misconceptions incidental to those who pursue success in the same or in neighbouring towns are by nothing more surely removed than by the easy intercourse of a friendly meeting. The reviewer and the reviewed sit down together at the social board in charity. Youthful and ambitious members of the profession, solicitous to leave nothing undone that may conduce to professional success, can on few other occasions see so many of the members of the profession whose career has been successful; and derive from an acquaintance with them the important conviction that, notwithstanding the general ignorance of the public, and the occasional prosperity of the unlettered and mendacious quack, those who most enjoy the public confidence most deserve it by their attainments and their studious labour, and do most honour to it by simplicity of manners and integrity of character. Divided, as medical friends for the most part are, by distance, and enchained by duty, there was long wanting some inducement strong enough to make them occasional truants from their daily round of duties; and such an inducement has arisen in the annual meetings of the Association, which we have each year observed to be attended by some of the busiest as well as the ablest men engaged in the practice of medicine and surgery. They indeed, above all others, must be sensible of the advantage of change of place, of air, and of ideas, and the exchange for social pleasures of the common and engrossing cares of life. It is impossible to look on the crowd of faces at these meetings without seeing indubitable signs of the habitual anxieties which so soon "delve the parallels" in the brow of those whose business it is to cure diseases; and without being convinced that to none is the occasional refreshment of a happy holiday more useful.

A lover of his race might derive satisfaction from repairing beforehand to the place of annual meeting, and watching the arrival of the coaches. From the interior or exterior of the Telegraph and Highflyer, he would behold emerging or descending many a wise and venerable looking gentleman, arrayed in black; wearing perhaps a somewhat portentous hat, and aided by a cane much respected in the localities where it is usually flourished. The waiters and chambermaids of the hotels are seen to stand amazed at the sudden influx of gentlemen of clerical aspect, saving their paleness and attenuation, and, accustomed as they are to the marks of men of fashion and men of commerce, are perplexed with a new race, to them collectively unknown, and whose individual gravity unbends, amidst the recognitions of the coffee-room, and becomes lost in the general joy of the newly assembled company. The greetings on the steps and in the passages are many and hearty; the exclamations, every time the door opens and a new arrival occurs, not a few; and the merriment great, on ascending to the fifth story, to find that to this height the dignity of a houseful of doctors has come at last. Many are the pleasant little dinner-tables made up, and pleasant, above all, is the talk. Here you have great men who have never met since both were pale and anxious students; here others, who have never seen each other since they were boys. When the two grave-looking men now discoursing by the window were last together, it was as apprentices in "a northern county, bounded on the east by the German Ocean," &c. &c., when, if the truth must be remembered, much of their time was employed, as in the case of Lord Thurlow and Cowper, when clerks to an attorney, "in giggling and making giggle." They giggle no more; but they smile yet, and are happier at this moment than they have been for years. You hear the names of Dr. Hawthorn and Mr. Primrose announced: the one was your schoolfellow, the other is your cousin; and you knew both as idle, happy, clever, careless boys; but have never seen them since boyhood. In Dr. H., now fifty years old, you recognize the stout, good-humoured face of him whose Latin exercises you used to perform for mere love; and in Mr. P. the thin and meditative youth,—so given to poetry, and so smitten in old time with one of the beauties in Miss Neversmile's boarding-school. Dr. H. is stouter than ever, although paler, and he is full of all sorts of learning, mixed with abundance of goodnature. Mr. P. is still pale and meditative; but writes no more sonnets: he has lost his health in studious researches, and has a wife and seven children. With these and many other friends you dine on this first evening, and you find all old feelings reviving which seemed dead. There is an unspeakable satisfaction in discovering that the good qualities of the boy have ripened into the virtues of the man; and that, whilst the intellect has undergone its highest cultivation, the heart has lost none of its warmth. The grace imparted to the mind by the liberal sciences connected with medicine polishes without destroying the gems disclosed in youth, and, with increased splendour, they retain all their intrinsic value.

Who shall describe the talk of this happy evening! In this secure corner we have a knot of eight Edinburgh graduates, of some twenty years' standing, or it may be more or less. Their talk is of old times; of Gregory and Duncan, of Rutherford and Home and Monro; of walks to Arthur's Seat, and of breakfasts at Roslin; and of epidemics watched in the clinical wards, and curious cases in Libberton's Wynd and the Bow; and of perils past in examinations, and of subsequent progress made by all; of difficulties overcome, opponents baffled, talents acknowledged, and merit rewarded.

Soon after the arrival of the members at the town of meeting, it is customary for them to repair to some central committee-room, to record their names in the book of arrivals. The preciousness of that book can only be known to those who, running through its crowded pages, have felt their breasts throb with delight on reading successively the names of much-loved friends, who have already reached the place, and are for a few days once more their actual neighbours. Intermixed with these occur the names of many distinguished authors and many practitioners of celebrity, whose fame has reached us, and whom we long to see. An hundred recognitions take place. Questions and replies, enquiries and congratulations, and heart-rejoicings abound; and every man feels that the cares which did but yesterday cling close to him, as if resolved to quit him never, are full a thousand miles off. Happy friendships, long-forgotten smiles, old talk, and, as it seems, youth itself, all are restored. Not but what we mark, in these meetings, the inevitable transition from youth to age in many a friend. Encroaching wrinkles, a sprinkling of silver hairs, a suspicious patch denuded over the organ of self-esteem, and a slight decay of animal joyousness: all these we cannot but discern, although they are matters seldom alluded to, and do but raise a slight suspicion that our friends recognize some signs that even we ourselves are not immortal. But, if the outward figure yields to the inevitable law, the mind has gained the while. We observe in many a friend the increase of knowledge and accomplishments, and greater serenity, the effect, probably, of the habitual contemplation of life and death, which is creative of an unpretending philosophy that gives a peculiar charm to the conversation and life of a medical practitioner of a reflective mind and amiable character. It is a proud thing to know, too, that amongst those whom we love for their good qualities, and respect for their knowledge, and admire for their sincerity, the greater part are men of mark and influence among their neighbours; and known as the supporters of every truly liberal doctrine, the promoters of every real improvement, the friends, in short, of all their fellow-creatures. Of all the assembled crowd, rejoicing in this day, devoted to friendship, to cheerful conversation, and refreshing leisure, there is perhaps not one whose mental or whose moral attributes do not diffuse their influence to some circle more or less extended of friends, neighbours, and acquaintance; and for the most part, without all question, for good.

### FIRST DAY'S MEETING.

The first meeting of the Council of the Association was held on the morning of Wednesday, July 19th, at the rooms of the Literary and Scientific Association of Cheltenham. The exterior of this building is classical and elegant, on the model of a temple at Athens. In it lectures are frequently delivered to the inhabitants and visitors of Cheltenham, and tend, we hope, to diffuse a love of science and letters in that fashionable and agreeable place of resort. The whole of the building was on this occasion given up to the members of the Association. Indeed, we must observe that the arrangements of the Cheltenham committee were in every respect so liberal and judicious, and dictated by such an evident desire to promote the comfort of those attending the meeting, as to reflect upon them the highest degree of credit. The chair was taken by Dr. Holme, of Manchester, at half-past twelve o'clock, and the Council meeting did not break up until half-past three.

About eight o'clock in the evening, the chair was again taken by Dr. Holme, and the general business of the meeting commenced. In a short speech, expressive of his sense of the honour done him by appointing him to preside at the meeting held last year at Manchester, Dr. Holme resigned the chair to the president elect, Dr. Boisragon. The respect of the members for Dr. Holme was strongly manifested towards him by the meeting, all justly feeling that his great learning and his high character, as well as his zealous exertions for the welfare of the Association, conferred honour upon them, and entitled him to their grateful remembrance.

Dr. Boisragon's speech on assuming the chair was, like that of the late Dr. Carrick, at the Bristol meeting, read from a manuscript; a plan which ensures correctness, and relieves the timidity of an unpractised speaker, but which, whilst it was not on this occasion requisite for the latter reason, (for Dr. Boisragon possesses remarkable fluency and ease of diction,) gives a dulness to the commencement of the proceedings which is not desirable. As written reports have generally to be received at these meetings, and an elaborate retrospective address, it would certainly be desirable that the other parts of the business of the meeting should not lose any possible portion of animation. Dr. Boisragon's address was by no means without animation. He dwelt, with evident complacency, on the rise and progress of a spa, with the celebrity of which his own has long been associated; and magnified the salubrity of Cheltenham beyond what some of the medical topographers who had descended to it that evening from the surrounding hills were inclined to believe it quite deserved. Dr. B. also introduced a witty but somewhat long exposition of the follies of homœopathy; and alluded feelingly to the deaths, since the last anniversary, of Drs. John Johnstone and Dr. Carrick, both of whom had presided on former occasions.

Dr. Hastings, then, as one of the secretaries of the Society, read the Report of the Council for the year 1837. There are not a few members of the Association who fancy that their professional engagements present an insuperable obstacle to their attending the anniversaries of the Association, who should reflect that this eminent practitioner, whose wide engagements in Worcestershire and the neighbouring counties are well known, has never yet been absent from one meeting, and that he finds time to attend during the year to all the business of the Association, in which few of those who enjoy the mere annual festival take any share. The results have been in proportion to the great exertions made by Dr. Hastings; although even these could not have raised the Association in five years from a commencement of sixty persons to a society of nearly one thousand, unless seconded by the general estimation he so justly enjoys, and the extensive influence he has thus been enabled to exercise. There can be no more gratifying spectacle than that of a physician so eminent and influential devoting all his leisure and all his influence to the noblest objects.

The Report stated that the number of members had, during the past year, increased from 600 to 940; chiefly in consequence of the establishment of District Branches. In this respect the Eastern Association has been honorably distinguished by abandoning all exclusive arrangements, and resolving itself into the general body; of course, reserving to itself the right to hold separate meetings, a right which it is much desired that each District Branch should exercise. For this arrangement we presume the Association is not a little indebted to Mr. Crosse, of Norwich, that distinguished surgeon being also one of the members who is, notwithstanding his extensive and even harassing engagements, among the most regular attendants at the annual meetings. District Branches have been established also at Wells, Bath, and Southampton, and the latter is already very extensive. The following resolutions were recited in the Report, as called for by the formation of these, and they were afterwards unanimously agreed to by the meeting.

"1. That, in order to fulfil more effectually the several purposes for which the Provincial Association was formed, it is expedient that a still more intimate union of its members be promoted, by the establishment of District Branches.

"2. That members of the Association be at liberty to form District Branches wherever it may suit their convenience.

"3. That, in order to facilitate the formation of such Branches, and maintain uniformity amongst them, the General Council provide suitable instruction for the guidance of those who may unite in instituting them.

"4. That conformity with these instructions be further ensured, by the initiatory proceedings, and organization of such Branch, being submitted to the General Council, for their revision and approval.

"5. That the District Branches be free to govern themselves as their respective members may think fit; but that the by-laws ordaining the special government be submitted to the General Council previously to their taking effect, in order to guard against the possibility of any such by-laws contravening the fundamental laws of the Association.

"6. That all members appointed to offices by the District Branches be forthwith enrolled as members of the General Council, on the appointments being officially notified to the General Council; it being highly expedient that all who engage in the executive management of the District Branches should be also members of the General Council.

"That the expenses incurred by the District Secretaries in conducting the proceedings of the District Branches be defrayed from the general fund, provided such expenses do not in any instance exceed one-seventh part of the guinea subscribed by each member enrolled in the District Branch.

"8. That, if any circumstances arise in the formation of District Branches which call for a larger expenditure than what is allowed by the foregoing resolution, such expenses, provided they do not exceed one-fourth of the guinea, may be allowed, by a statement of the circumstances being made known to the General Council."

Of the finances, the Report contained a satisfactory account. During the past year the receipts have been 1,010*l.* 8*s.* 11*d.*, and the expenditure 698*l.* 6*s.* 10*d.*, leaving a balance of 312*l.* 2*s.* 1*d.* The chief items of expenditure are the publication of the annual volume of Transactions, and of a Poor-law Report made by a committee appointed at Manchester.

The Council complain of the apathy of provincial physicians and surgeons attached to hospitals and infirmaries, as regards Vital Statistics. They also notice the question of Parochial Medical Relief; the proceedings of the committee of the Benevolent Society; and the new Act relative to the Registration of Diseases, in connexion with which subject they exhort the members to supply Meteorological Reports. They also announce that Dr. Thackeray, of Chester, "has increased his already liberal donation to fifty pounds, to be given as a prize for the best essay on a medical subject." This prize the Council propose to call the Thackeray Prize, "to be open to the competition of the members of every accredited school for medicine and surgery in the United Kingdom."

Several resolutions, most of which were suggested by the Report of the Council, were then submitted to the meeting, and, after discussion, were generally passed. It was unanimously resolved that the next anniversary meeting should be held at Bath, under the presidency of Dr. Barlow; and that Dr. Malden, of Worcester, should deliver the Retrospective Address. Committees were appointed for various objects; among others, "to decide on the subject of the Thackeray prize;"—"to watch over the interests of the profession at large, and suggest such measures as may appear necessary to meet circumstances as they arise;"—"to report on the communications received respecting the Influenza," &c.; and resolutions were passed, pledging the gentlemen present to "urge upon the members of the legislature, in their several localities, the importance of an enlightened consideration of the questions touching the public health pending in Parliament," and suggesting to the members of the Association generally "the propriety of lending their aid to carry into effect the recent Act to procure an improved Registration of Births, Deaths, and fatal Diseases."

### SECOND DAY'S MEETING.

Although the business of the preceding evening was not brought to a conclusion until a late hour, the gardens of the magnificent Pitville Spa were full of medical stragglers early the next morning, a part of the plan being a public breakfast in the spacious rooms above the grand pump-room of that establishment. Many acres of ground surrounding this spa, which lies to the East of Cheltenham, and about a mile from High-street, are laid out with much taste and beauty. Fine trees, a beautiful piece of water, numerous shaded alleys, and a building which might almost be called a palace, commanding splendid views, contribute to the attractions of the spot, around which are scattered many elegant mansions, intermixed with comfortable houses of more moderate pretensions. At nine o'clock, the whole party sat down to an excellent breakfast, provided under the direction of Mr. Seymour, the resident at the Spa. At the conclusion of the repast, and by the particular desire of many of the subscribers to a portrait of Dr. Hastings, proposals for which were placed before the members at the Manchester meeting, Dr. John Conolly communicated to those present that the design would now be proceeded in forthwith; and papers were sent round to receive additional names. The intention of the subscribers is to present Mrs. Hastings with a portrait of Dr. H., painted by an eminent artist, each subscriber being furnished with a finished engraving from it. No compliment was ever better deserved; and none, we should imagine, could be thought of more likely to gratify Dr. Hastings' family.

The members of the Association then adjourned to the Literary and Scientific Institution, where Dr. Marshall Hall, (who had travelled from London with Dr. Webster of Dulwich to meet the association,) delivered a highly interesting lecture to them on the Excito-Motory System of Nerves, illustrated by a few experiments. All the hearers bore willing testimony to the pleasing and candid manner of the lecturer, as well as to his zeal, industry, and ability; and a vote of thanks to him was carried by acclamation.

At twelve o'clock a general meeting of the members took place in the Montpelier Rotunda, a large and elegant circular apartment, well known to all who have drunk the Cheltenham waters. Commodious apartments at the side of the large room were appropriated to the Poor-Law and other Committees. Here, as at the rooms of the Literary and Scientific Association, the arrangements for the reception and comfort of the members met with universal approbation.

The proceedings of the day were opened by Dr. Boisragon, the President, and the first business was to move an address from the Association to the Queen. The address was proposed by Dr. Barlow and seconded by Dr. John Conolly, and was unanimously adopted by the meeting. It was resolved that the address should be presented by a deputation from the Association consisting of Dr. Boisragon the President, Sir Astley Cooper, Dr. Kidd, Dr. James Clark, Dr. Hastings, and the mover and seconder.

Dr. James Lomax Bardsley, of Manchester, being then called on by the President to read the Retrospective Address, delivered a luminous and elaborate discourse, the length of which, and the numerous and minute details and elaborate research displayed in it, defy the abridgment indispensable in a sketch of this nature. It comprehended all the discoveries and improvements of the past year, in Anatomy, Physiology, Phrenology, Medical Statistics, Medical Literature, and Chemistry; took a view of the nature, causes, and treatment of Cholera and Influenza; paid a just tribute of respect to the memory of those departed lights of science, Dr. Henry, Dr. Turner, Dr. John Johnstone, and Mr. Ransome, of Manchester; sketched out the most advantageous plan for the education of professional men, and triumphantly exposed the vices and follies of ignorant quackery, stating very justly that licentious empiricism was strangely at variance with the boasted intelligence of the nineteenth century, but that charlatanism seemed to receive encouragement in proportion to the boldness and impudence with which it was promulgated. The address was most enthusiastically received, and it was proposed by Dr. Forbes, and seconded by Dr. Symonds, that it should be printed and circulated among the members.

Dr. Baron, being now called on by the Chairman, read the Report of the Benevolent Committee, and prefaced it by some remarks as to the object that Committee had in view, which was to relieve members who had fallen into unavoidable misfortunes. The report fully explained these intentions, and stated that they had already, in some instances, been carried into effect.

It was then moved by Dr. Holme and seconded by Dr. Wm. Conolly, "that the Report be adopted and printed; and that the Association take this opportunity of expressing their thankfulness that the Benevolent Fund has been rendered useful to some of their suffering brethren; and that the suggestions contained in the Report for increasing and collecting the contributions, be earnestly recommended to the consideration of every Member of the Association."

Dr. Davies, of Presteign, in a highly flattering manner, proposed that their visitor, Dr. Macartney, Professor of Anatomy, Trinity College, Dublin, be elected an Honorary Member of this Association, which being seconded by Mr. Crosse, of Norwich, was carried by acclamation.

The Secretary, Dr. Hastings, then read the letter of Dr. Thackeray, announcing the liberal prize of 50*l.* for an Essay on a Medical Subject; and on the report of the Committee on that point being called for, Dr. Barlow solicited an extension of the time allowed them to two months, and recommended that the Essays for competition should not be sent in till May, 1839.

Dr. Webster, of Dulwich, seconded by Dr. M'Cabe, proposed "that the highly important report of the Poor Law Committee be read," which was accordingly done by Mr. W. H. Rumsey, of Chesham, Secretary to that Committee, as follows:—

"The Poor Law Committee having been requested to direct their attention once more to the state of medical relief for the sick poor, submit the result of their deliberation for the consideration of the Association. Seeing that the evils detailed in the report of last year continue unabated, notwithstanding the steps already taken by the Association and the profession at large, your Committee think it highly important at the present juncture that energetic measures should be pursued, and beg to urge this question on the attention of the Legislature and the public, until a parochial or national system of medical relief for paupers be settled on a basis equally humane to the poor and just to medical practitioners. With a view effectively to make known the opinions and feelings of this Association, your Committee also recommend that personal and written communications be made by members of the Association, in every locality, to their respective representatives in Parliament. Your Committee also recommend that petitions be presented immediately on the assembling of Parliament, praying for a special, full, and impartial enquiry into the subject of medical relief, and for the production of official returns of all medical contracts made under the new law, of the number of practitioners employed compared with those under the former system, of the extent of districts intrusted to medical officers, of the amount of their salaries, the mode of appointment, the number of patients attended and visits made by each medical attendant. If after the adoption of these measures there be not a fair prospect of redress by Parliament, it is the opinion of your Committee that members of this Association ought no longer to sanction a system alike degrading to themselves, and cruel and delusive to the sick poor. To use the words of their last report, 'We ought firmly to decline any participation in the medical regulations under the New Poor Law.'

"If the profession had been true to itself, and if medical men had in private acted up to those declarations which they have made in public, the matter would have long ago been equitably arranged; but whilst unprofessional, mean, and selfish conduct continues to disgrace our body, we cannot wonder that the authorities should take advantage of our delinquencies.

"The influence of this Association ought to be exerted upon its members, to induce them not to swerve from those admirable principles upon which we profess to move.

"Your Committee, in thus bringing to a conclusion their labours, cannot refrain from expressing a confident hope that the subject which has for so long a period engaged their attention, will be followed up to a successful issue by the energies and decision of the Association; and they view with much satisfaction the appointment which has just been made of a permanent Committee of this Association, "for the purpose of watching over the interests of the profession," convinced that this subject will immediately engage their entire attention."

Dr. Webster then moved, and Mr. Addison of Malvern, seconded the resolution—"That a petition be drawn up in conformity with the recommendations of the Poor Law Committee, to be signed by the President on behalf of this Association, and presented to both Houses of Parliament immediately on their assembling."

It was also moved by Dr. Malden, and seconded by Dr. Coley—"That a form of letter in conformity with the recommendations of the Poor Law Committee be forthwith prepared and printed and transmitted without delay by every Member of this Association to their parliamentary representatives and friends;" and, on the motion of Dr. W. Conolly, "A vote of thanks was passed to the Poor Law Committee, especially to Mr. Rumsey, for their important and invaluable services."

After the breaking-up of this interesting meeting, all the proceedings of which were marked by the utmost unanimity of feeling, the members had about an hour and a half of leisure before the hour of dinner; and were to be seen dispersed in groups in the beautiful gardens of the Montpellier Spa, and in most of the principal streets of the town, particularly in High street, where their numbers and appearance caused evident surprise among the indolent occupiers of the green chairs in front of the libraries, and, we fear, deluded many an anxious tradesman into the pleasing belief of a remarkable arrival of invalids. It was truly agreeable to see the numerous introductions, the friendly groupings, the lively countenances of so many intelligent and laborious members of a cultivated and hard-working profession.—But time advances with steady pace, and even dinner is at length ready. This entertainment was laid out in the Assembly Room, certainly one of the finest apartments in the kingdom; spacious, lofty, well-proportioned, decorated with singular taste and elegance, and so arranged as to preserve, even with a large company, the comfort of a moderate and equal temperature. We have never seen a dinner more comfortably served. Most of the public dinners in London which we have witnessed were held in immense and dingy apartments, and hurried over with numerous discomforts. We have been constant in our attendance at the meetings and dinners of the Association. That of Bristol left indelible impressions, as was to be expected in a city so renowned for luxurious hospitality. That of Birmingham will never be forgotten, for every opposite quality. That of Oxford was befitting a place scarcely less celebrated for its magnificent entertainments than for its learning and science. That of Manchester was substantially good and comfortable. But the palm of dinner must be conceded to Cheltenham, where the abundance and goodness of the dinner itself, its cheapness, the excellence of the wines, the beauty of the apartment, and the delightful accompaniment of the first quadrille band out of London, conspired to make every guest forgetful of the admirable precepts propounded often to obedient and disobedient patients. Of such precepts, however, truth to say, no men are less forgetful than medical men; and the example of their well-restrained and rational conviviality might be usefully followed by other classes of people. We are not among those who consider it unphilosophical, in the ordering of these meetings, to take heed to the substantial refreshments. Many a good institution has suffered by the insufferable badness of the annual dinner at a common inn; and, for the good of the District Branches, as well as of the parent stock, we think it by no means unworthy to be recorded that an excellent dinner was provided at Cheltenham for ten shillings and sixpence a head, and that the wines, being furnished by a respectable wine-merchant, were pure and good, and sold at reasonable prices. The thanks of the company were certainly most due to the committee by whom such excellent arrangements were carried into effect. The comfort resulting from it was unspeakable, and most felt by those in the habit of paying one guinea for the privilege of being half starved and half poisoned.

We regret that we cannot afford space for full details of the dinner. Dr. Boisragon presided, and, it is almost superfluous to say, with urbanity, cheerfulness, and dignity, and did all that any president could do to ensure the enjoyment of every one present. Nothing could be more felicitous than the manner in which he proposed the health of Dr. Hastings; of whom he most justly observed, that he had "created an era in the annals of the profession, and had accomplished more than had been done by any individual since the days of Linacre. Eminent for his attainments in literature and science, he was endeared to them as the originator and founder of that

Association, which would do much to redeem the medical profession from the calumnies of those who preferred ignorant arrogance, and impudent empiricism, to talent, learning, and self-denial."

To those of our distant readers who have not the happiness to be acquainted with Dr. Hastings, and who yet receive these details with interest, we wish we could convey an adequate impression of the spirit and energy of his reply to these demonstrations of regard. Dr. H., although he has attained the highest degree of professional celebrity, is still in the prime of life. To great talents, and an industry which seems incapable of being exhausted, he adds the fervour of temperament, which, when, as in his case, united with a calm judgment, leads to the undertaking and execution of great and noble designs; and these qualities are associated with a highly benevolent disposition, and the most frank, cordial, and unaffected manners. It is easy to perceive, by the terms on which he is with his immediate medical neighbours, (many of whom invariably accompany him to the meetings of the Association,) that he is in the habitual practice of the utmost professional liberality; and the extent to which he enjoys the confidence of the public is attested by a very extensive practice, and by the power he has so admirably shown that he possesses, to secure the prosperity of many local institutions of great utility to the part of England in which he resides. His speech in acknowledgment for his health being drunk abounded with warm and generous feelings, and was distinguished by a freedom of sentiment on all points affecting the state of the profession which ought to secure both him and the Association from the animadversions of a portion of the medical press, which we can only ascribe to complete misconception of his views and those of the majority of the members, and especially of those who are generally the most active in its affairs.

When Cheltenham was first proposed as an eligible place for holding the late annual meeting in, some of the members of the Association openly expressed their doubts whether, in a watering place, devoted to pleasure and to fashion, the medical residents would be found to care anything about the Association or general professional interests. It is, therefore, particularly due to Cheltenham to state, that the manner in which the Association was received there was no less gratifying to the members than honorable to that town, and to the character of its medical residents. Among these, the members sufficiently testified, by their cordial reception of Dr. Baron, when that learned physician came forward at the morning meeting to advocate the claims of the Benevolent Fund; and again, after dinner, when they drank his health as the biographer of Jenner, (once a resident of Cheltenham,) that they considered him to hold a very high place: and it was not a little gratifying to hear one so distinguished by acquirements and character speaking with fervent admiration of the great discoverer of vaccination, whom he had personally known, as "not only one of the greatest of medical men, but one of the best of men; one who to him (Dr. B.) had been as a father and a friend, and than whom a kinder, more just, and more benevolent and single-hearted man never existed." Such is, indeed, the true description of the moral qualities of a good physician; and it will ever be a source of pleasurable reflection to us to have heard this surviving testimony to the illustrious Jenner, so many years after the grave has closed upon his exertions for the benefit of mankind.

We could dwell with satisfaction on the manner in which the president proposed, and the company received the healths of Dr. Bardsley, of Dr. Holme, Dr. Barlow, and other eminent members of the Association; men whose very names, in all who know them, excite recollections of talents and virtues which command equal respect and affection. The manner in which Dr. Barlow's health was received sufficiently evinced the consideration with which that learned physician is regarded by his brethren. In assembling under his auspices at Bath, next July, we feel assured that the members will experience more than ordinary pleasure, arising out of a regard for so enlightened a president and so good a man. To become acquainted with such men is a privilege which many will ever consider as one of the most cherished that the Association has conferred or can confer upon them.

To conclude our notice of this agreeable day without allusion to Dr. Malden's incomparable after-dinner speech, would argue a want of appreciation of the wisdom and the wit which that highly respected member of the Association has the art of blending in a manner quite unapproachable by the generality of speakers. Relying on the possession of extensive and solid acquirements, and perhaps a little on a prepossessing exterior, Dr. Malden ventures upon flights of fancy and of mirth which take philosophical men by surprise, and shake the sides of grave and gay alike. When you think he is venturing into altitudes whence there is much danger of a lame and lamentable fall, he spreads out a parachute of eloquent good sense, and descends gracefully to a less elevated region: and, when men of less smartness and readiness rejoice that he is treading upon level ground with them, he extends his light wings, and leaves them gaping and amazed, and, withal, amused to the last degree. His language is figurative and startling: ancient learning, modern accomplishments, forcible imagery, classical allusion, are all at his command. We venture to prophesy that the Retrospective Address to be next year delivered by him, will justify all that we have said of his attainments, and illustrate our description of those graces of manner which have rendered him so especial a favorite with his friends and acquaintances.

When all the toasts had been gone through, a Conversazione was held in an adjoining room, and, although of the intercourse there facilitated, and the interchange of experience and of friendly sentiments, no public record can exist, that last friendly hour had many and great attractions, which disposed, we feel quite sure, many who then parted, not to forego future opportunities of renewing the same social and elevated enjoyments another year. In a word, whatever of good feeling, whatever of the spirit of high and legitimate research, whatever of desire for the general good of the profession, its best friends would wish to see encouraged, received at this great meeting of physicians and surgeons all the support which their example and their expressed opinions could give to such objects. That the best interests of the profession will be promoted and defended, and numerous useful investigations suggested, in consequence of the existence of this large and influential Association, we do not entertain a doubt; but, if no such benefits were to follow in a formal shape from its establishment, we shall still consider that its Anniversaries are calculated to produce effects the most beneficial on the general tone and temper of the profession; and, with this fixed impression, we heartily pray that its duration and its prosperity may be as extended and as brilliant as its distinguished founder and as its warmest supporters can desire.

---

